# Sol–Gel-Synthesized Metal Oxide Nanostructures: Advancements and Prospects for Spintronic Applications—A Comprehensive Review

**DOI:** 10.3390/gels11080657

**Published:** 2025-08-19

**Authors:** Kais Iben Nassar, Sílvia Soreto Teixeira, Manuel P. F. Graça

**Affiliations:** I3N-Aveiro, Department of Physics, University of Aveiro, 3810-193 Aveiro, Portugal; silvia.soreto@ua.pt (S.S.T.); mpfg@ua.pt (M.P.F.G.)

**Keywords:** sol–gel synthesis, spintronics, metal oxide nanostructures, transition metal oxides, condensed matter physics

## Abstract

Spintronics, an interdisciplinary field merging magnetism and electronics, has attracted considerable interest due to its potential to transform data storage, logic devices, and emerging quantum technologies. Among the materials explored for spintronic applications, metal oxide nanostructures synthesized via sol–gel methods offer a unique combination of low-cost processing, structural tunability, and defect-mediated magnetic control. This comprehensive review presents a critical overview of recent advances in sol–gel-derived magnetic oxides, such as Co-doped ZnO, La_1−x_Sr_x_MnO_3_, Fe_3_O_4_, NiFe_2_O_4_, and transition-metal-doped TiO_2_, with emphasis on synthesis strategies, the dopant distribution, and room-temperature ferromagnetic behavior. Key spintronic functionalities, including magnetoresistance, spin polarization, and magnetodielectric effects, are systematically examined. Importantly, this review differentiates itself from the prior literature by explicitly connecting sol–gel chemistry parameters to spin-dependent properties and by offering a comparative analysis of multiple oxide systems. Critical challenges such as phase purity, reproducibility, and defect control are also addressed. This paper concludes by outlining future research directions, including green synthesis, the integration with 2D materials, and machine-learning-assisted optimization. Overall, this work bridges sol–gel synthesis and spintronic material design, offering a roadmap for advancing next-generation oxide-based spintronic devices.

## 1. Introduction

In recent decades, the rapid evolution of condensed matter physics, nanotechnology, and materials chemistry has fostered significant advances in functional oxide research [1,2,3]. These developments have been driven by the increasing demand for high-performance electronic, magnetic, and multifunctional devices [4,5,6]. Within this context, spintronics has emerged as a transformative paradigm that exploits the spin degree of freedom in addition to the charge, enabling new concepts in non-volatile memory, logic circuits, quantum information processing, and multifunctional sensing [7,8,9,10]. The progress in this field has been tightly linked to advances in thin film growth, nanostructuring, and defect engineering strategies [11,12,13]. Among the various synthetic methods, sol–gel chemistry has attracted particular interest for producing complex oxide nanostructures with controlled compositions, morphologies, and defect states at low processing temperatures [14,15,16,17,18,19]. This versatility allows for the fabrication of dilute magnetic semiconductors, ferrites, and perovskite oxides tailored for spin-polarized transport, magnetoresistive effects, and magneto-optical coupling [20,21,22,23,24,25]. Furthermore, the integration of sol–gel-derived oxides into device architectures has benefited from the technique’s scalability, substrate compatibility, and ability to incorporate diverse dopants [26,27,28,29,30]. Recent studies have also demonstrated the feasibility of combining sol–gel synthesis with advanced characterization and computational modeling to optimize the spintronic performance through the precise control of microstructures and electronic structures [31,32,33,34]. These trends underscore the interdisciplinary nature of modern spintronics, which draws upon solid-state physics, surface science, chemical processing, and device engineering [35,36,37,38], while also aligning with broader technological priorities such as energy-efficient computing, flexible electronics, and multifunctional sensor platforms [39,40,41,42,43,44,45].

Spintronics, or spin electronics, is an emerging field that exploits both the spin and charge of electrons in solid-state systems. Unlike conventional electronics, which rely solely on the electron charge, spintronics enables new paradigms in data storage, sensing, and quantum computing through the manipulation of spin polarization and magnetic ordering. Among various synthetic approaches, the sol–gel method has gained attention due to its ability to produce metal oxide nanostructures with a controllable size, shape, and chemical homogeneity [46,47,48,49,50,51,52,53,54]. Sol–gel chemistry, whether hydrolytic or non-hydrolytic, offers low-temperature processing routes ideal for designing oxide-based ceramics, like zirconia, aluminum titanate, and mullite, with enhanced structural and electronic properties [46,47,48,49,50,51,52,53]. Furthermore, understanding precursor interactions, gelation dynamics, and crystallization pathways provides a foundational basis for tailoring nanoscale architectures for high-performance applications [48,49,50,51]. These advancements are particularly relevant to next-generation spintronic devices, where the electron spin and charge are exploited for multifunctional behavior, data storage, and quantum computing potentials. The realization of spintronic devices critically depends on materials that exhibit robust spin polarization, long spin lifetimes, and, often, room-temperature ferromagnetism. Traditional materials like permalloy and Heusler alloys have limitations in their cost, scalability, and compatibility with semiconducting platforms [55,56,57].

In contrast, metal oxide semiconductors and ferrites, such as ZnO, TiO_2_, Fe_3_O_4_, NiFe_2_O_4_, and La_1−x_Sr_x_MnO_3_, are emerging as promising candidates due to their abundant availability, tunability, chemical stability, and compatibility with existing complementary metal oxide semiconductor (CMOS) processes [58,59]. Spintronics, or spin electronics, refers to the study and application of the electron spin and its associated magnetic moment in solid-state devices, offering new paradigms for data processing beyond charge-based electronics. Key physical phenomena underlying spintronic functionality include spin polarization (the alignment of electron spins), the spin injection and detection across interfaces, spin transport and relaxation mechanisms, magnetoresistance effects (such as giant and tunneling magnetoresistance), and spin–orbit coupling. These mechanisms enable critical applications in non-volatile memory, magnetic sensors, and quantum computing. For practical device implementation, materials must exhibit a robust spin polarization, a long spin coherence, and preferably room-temperature ferromagnetism. As discussed in this review, sol–gel-synthesized magnetic metal oxide nanostructures offer a promising platform to engineer these properties by tailoring the dopant incorporation, defect profiles, and nanostructure morphology through molecular-level chemical control.

**Figure 1:**
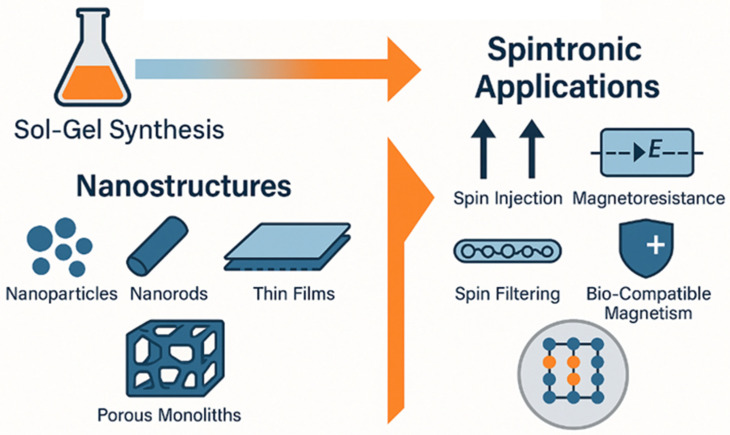
Schematic overview: Sol–gel synthesis pathways and nanostructured oxide architectures for spintronic technologies.

Importantly, the synthesis method profoundly affects the material’s magnetic, structural, and electronic properties. Among various routes, sol–gel processing has emerged as a powerful, low-temperature, and scalable technique for the synthesis of nanostructured oxides with a tailored composition and morphology [60,61,62,63]. It allows for excellent control over the dopant distribution, nanoparticle size, phase purity, and interface properties, all of which critically influence the spin-dependent transport characteristics of the resulting material. The sol–gel process, based on the hydrolysis and polycondensation of metal alkoxides or salts, is especially attractive because it can yield thin films, nanoparticles, aerogels, and porous monoliths. Furthermore, it allows for the incorporation of magnetic dopants (such as Co^2+^, Fe^3+^, and Mn^2+^) at the molecular level, resulting in homogeneous doping and, often, enhanced magnetic functionality [64,65,66,67]. This fine control is vital for tuning room-temperature ferromagnetism, a central requirement for practical spintronic devices. Another advantage of sol–gel processing lies in its versatility and low cost. Unlike physical vapor deposition techniques, sol–gel routes require relatively simple equipment and can be used to fabricate materials on flexible substrates, large areas, or in complex geometries. This scalability makes sol–gel synthesis particularly attractive for emerging flexible spintronic applications and multifunctional device architectures [68,69,70,71,72,73,74]. As visualized in Figure 1, sol–gel synthesis provides a bottom-up, tunable pathway to spin-functional materials. The ability to engineer defects, control the dopant distribution, and tune the morphology on the nanoscale is particularly advantageous for fabricating materials that exhibit room-temperature ferromagnetism, spin-filtering effects, and magnetoresistive behavior. Furthermore, sol–gel-derived thin films and nanostructures are highly compatible with lithographic and solution-based device processing, which is critical for scalability and cost-effective production [75,76,77,78,79].

However, challenges remain. Sol–gel-derived nanostructures often suffer from issues like secondary phase formation, dopant clustering, and reproducibility problems, which can obscure the intrinsic magnetic properties and complicate the interpretation of spin-dependent transport characteristics [80,81,82,83,84,85]. Moreover, distinguishing true ferromagnetism from defect-induced magnetic ordering or superparamagnetic behavior requires rigorous structural and magnetic characterization. The interaction between dopants, defects, and the host lattice must be understood both theoretically and experimentally to realize a reliable and reproducible device performance [86,87,88,89]. Recent advances in characterization tools, such as high-resolution transmission electron microscopy, X-ray photoelectron spectroscopy, Mössbauer spectroscopy, SQUID magnetometry, and electron spin resonance, have shed new light on these complex interactions, enabling better correlations between the structure and magnetism at the nanoscale [90,91,92].

This review aims to offer a comprehensive yet focused overview of the intersection between sol–gel chemistry and spintronic material design, with an emphasis on metal oxide nanostructures. We begin by outlining the principles of sol–gel synthesis and its application in fabricating key oxide systems. Next, we explore the structure–property relationships that govern spintronic behavior, highlighting the recent progress on materials such as Co-doped ZnO, Fe_3_O_4_, NiFe_2_O_4_, Mn-doped TiO_2_, and perovskite manganites. Finally, we address the critical challenges facing the field and suggest future directions for integrating sol–gel-based oxides into scalable, high-performance spintronic devices.

## 2. Sol–Gel Synthesis of Magnetic Oxide Nanostructures

### 2.1. Overview and Motivation

The sol–gel method has emerged as a central synthesis strategy for fabricating nanostructured metal oxides, especially in fields that require a low-temperature, scalable, and compositionally precise fabrication [93]. In the context of spintronics, where magnetic behavior and electronic transport are highly sensitive to the defects, dopants, and phase purity, the sol–gel route offers unparalleled control at the molecular level. Unlike conventional solid-state methods that typically require prolonged high-temperature processing and suffer from diffusion limitations, the sol–gel technique exploits solution-phase chemistry to enable the atomic-scale mixing of multiple cationic species. This leads to homogeneous doping, low-temperature crystallization, and better phase control in complex oxide systems.

**Figure 2:**
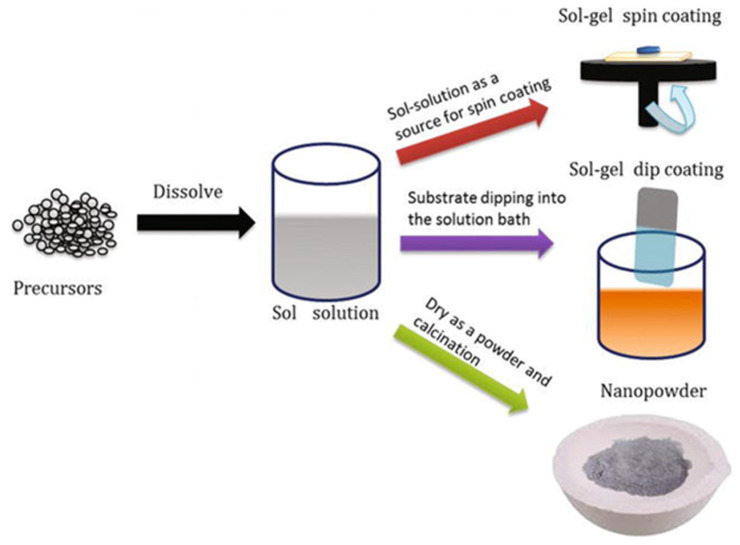
Schematic diagram of sol–gel processing [94].

Figure 2 schematically illustrates the sol–gel synthesis route starting from precursor materials [94]. These precursors are dissolved to form a colloidal suspension of particles in a liquid medium. This sol acts as a versatile source material for fabricating nanostructures through various downstream processes. One common technique is spin coating, where the sol solution is rapidly spun on a substrate to form a thin and uniform gel layer. This method is especially useful for fabricating thin films with precise thickness control, which is important for device applications in spintronics. Another approach is dip-coating, in which the substrate is immersed into the sol solution and then withdrawn at a controlled rate. This process deposits a uniform gel layer on the substrate and can be used to create coatings with different thicknesses and morphologies. Lastly, the sol can be dried and calcined to form nanopowders. Calcination at elevated temperatures removes organic residues and promotes crystallization, resulting in pure-phase oxide nanoparticles with a controlled size and morphology.

For spintronic applications, such as magnetic semiconductors, half-metals, magnetoresistive sensors, and tunnel junctions, the electronic and magnetic properties are extremely sensitive to the nanostructure, grain boundaries, and defect states [95,96]. The sol–gel approach offers the ability to precisely tailor these features through the control over the precursor chemistry, reaction conditions, and thermal treatment. This is particularly valuable for the synthesis of dilute magnetic semiconductors and magnetically ordered oxides, where parameters like the oxidation state, spatial distribution, and coordination environment of magnetic dopants (e.g., Co, Mn, Fe) strongly influence the occurrence of room-temperature ferromagnetism [97].

Moreover, the sol–gel method allows for the fabrication of a wide variety of morphologies and dimensionalities, including nanoparticles, nanorods, thin films, and porous 3D structures. Its compatibility with templating and soft lithographic techniques further expands its utility in device fabrication and integration. Despite its strengths, the sol–gel process is not without challenges. Issues such as secondary phase formation, variable doping efficiency, and uncontrolled defect structures can limit reproducibility and functional performance. Furthermore, the drying and calcination stages may introduce stress or result in shrinkage/cracking in thin films, making uniform large-area deposition more complex. Below is a comparative summary of the main advantages and disadvantages of the sol–gel method in the context of spintronic materials [98]. This foundational understanding of the sol–gel process forms the basis for the targeted design of functional magnetic oxides. In the following sections, we explore how these chemical principles translate into nanostructured materials with tailored properties for spintronic applications. Table 1 summarizes the advantages and disadvantages of the sol–gel method for materials.

To provide a broader context, Table 2 presents a comparative analysis of sol–gel synthesis with other commonly used fabrication methods for spintronic oxide nanomaterials, highlighting their respective advantages in terms of their temperature requirements, dopant control, scalability, and relevance to spintronic applications.

### 2.2. Fundamentals of the Sol–Gel Process

The sol–gel process is a versatile, low-temperature technique that enables the fabrication of advanced materials, particularly metal oxides, with controlled nanostructures. It involves a series of solution-based chemical reactions that convert molecular precursors into solid materials. This approach is particularly attractive for spintronic materials because it offers molecular-level homogeneity, a precise dopant distribution, and the potential for low-cost, large-area deposition [102,103]. The preparation of precursors: The sol–gel process begins with the preparation of suitable precursors, typically metal alkoxides or inorganic salts. Metal alkoxides such as titanium isopropoxide, tetraethyl orthosilicate (TEOS), and aluminum sec-butoxide are frequently used due to their reactivity and ability to form oxide networks upon hydrolysis. Inorganic salts such as nitrates and acetates can also be employed, especially when the cost, stability, or water solubility is a concern. The choice of the precursor directly influences the reaction kinetics, final composition, and properties of the nanostructured product [104].

Hydrolysis and condensation: The key chemical transformations in sol–gel synthesis are hydrolysis and condensation. In the hydrolysis step, metal alkoxides react with water to form metal hydroxides. This step can be catalyzed by acidic or basic conditions to modulate the reaction rate. In the subsequent condensation step, these hydroxide species link together through the formation of M–O–M bonds, forming a three-dimensional oxide network. The degree of condensation affects the gel’s porosity, homogeneity, and final crystallinity. The control over the pH, temperature, water-to-alkoxide ratio, and solvent system is crucial for tailoring the microstructure [105,106]. Gelation and aging: As the sol evolves into a gel, a continuous solid network forms throughout the liquid phase. This gelation process marks the transition from a liquid-like sol to a solid-like gel. During the aging period, the gel continues to develop, undergoing syneresis (shrinkage due to solvent expulsion) and further condensation reactions. Aging affects the porosity, mechanical stability, and degree of the network connectivity of the gel. Extended aging times can improve structural ordering and facilitate phase development at lower temperatures [107]. Drying and calcination: Drying removes solvents from the gel, which can lead to shrinkage, cracking, or the collapse of the porous network if not carefully controlled. Supercritical drying or freeze-drying are sometimes used to preserve porous structures. Calcination, or a thermal treatment, serves to decompose residual organics and crystallize the oxide phase. Parameters such as the heating rate, atmosphere, and maximum temperature directly affect the final phase purity, grain size, and defect structure. For spintronic oxides, calcination also determines the magnetic ordering, dopant distribution, and oxygen vacancy concentration [108].

The sol–gel process offers remarkable flexibility in engineering nanostructured materials across different dimensional regimes, enabling researchers to tailor the physical properties of oxides for spintronic and multifunctional applications. Zero-dimensional (0D) nanoparticles, for instance, are synthesized as isolated, quasi-spherical domains typically ranging from 2 to 20 nm. These nanostructures are particularly attractive for use in quantum dots, where quantum confinement effects dominate, and in biomedical applications, such as magnetic resonance imaging contrast agents, due to their high surface area and size-tunable magnetic response. Moving to higher dimensions, one-dimensional (1D) nanostructures, including nanorods and nanowires, provide a directional anisotropy that is advantageous for studying spin transport phenomena. Their elongated geometries facilitate coherent spin propagation and are therefore explored in applications like magnetic nanowire memory and nanoscale spin filters [109,110].

**Figure 3:**
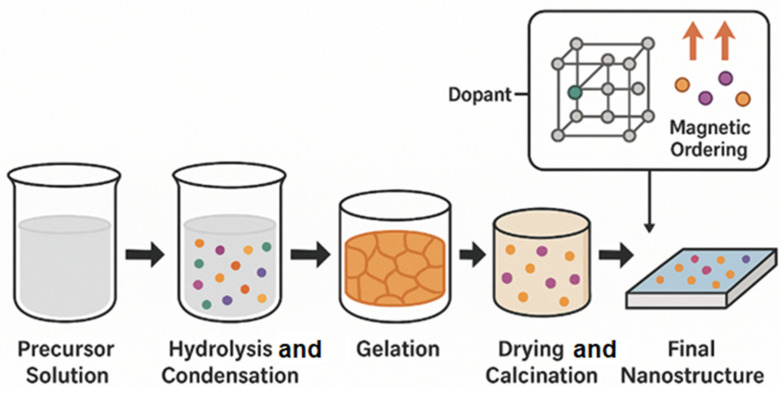
A schematic illustration of the sol–gel synthesis process for magnetic oxide nanostructures.

At the next level, two-dimensional (2D) thin films are perhaps the most critical form factor for the integration into functional spintronic devices. These films, often prepared via sol–gel spin coating or dip-coating methods, allow for the precise control over the thickness, crystallographic orientation, and surface roughness. Their compatibility with semiconductor substrates makes them ideal for applications in magnetic tunnel junctions, field-effect transistors, and magnetoelectric heterostructures. Lastly, the sol–gel process can also be extended to fabricate three-dimensional (3D) porous monoliths and aerogels, which exhibit interconnected pore networks and ultra-high surface areas. These 3D structures are not only useful for catalytic and sensing applications but also open new avenues in multifunctional devices where magnetism is coupled with ionic or electronic transport through porous matrices. Collectively, these dimensional variations underscore the sol–gel method’s extraordinary versatility in tailoring structure function relationships in advanced magnetic oxide systems [111]. Figure 3 shows the key stages: precursor solution preparation, hydrolysis and condensation, gelation, drying and calcination, and the formation of the final nanostructure. An inset highlights the dopant incorporation and the resulting magnetic ordering at the atomic scale.

### 2.3. Sol–Gel Variants and Techniques for Magnetic Oxides

The fundamental sol–gel process can be adapted in several ways to suit the synthesis of complex magnetic oxide nanostructures. Each variant offers distinct advantages in terms of particle size control, the dopant distribution, and scalability [112].

Alkoxide-based sol–gel: Alkoxide sol–gel methods utilize metal alkoxides that readily hydrolyze and condense under ambient conditions. This variant is particularly suited for the synthesis of simple oxides like TiO_2_, ZrO_2_, and Al_2_O_3_. For example, titanium isopropoxide can be hydrolyzed in an ethanol water mixture to form titania nanoparticles. The reaction conditions can be tuned to yield desired morphologies and phases (anatase vs. rutile). When doped with magnetic ions such as Co or Fe, these systems exhibit room-temperature ferromagnetism due to defect-mediated exchange interactions. The fine control over the stoichiometry and crystallization makes the alkoxide sol–gel an ideal choice for dilute magnetic oxide semiconductors [113,114].

The citrate–nitrate (Pechini) method: The Pechini method involves the formation of a polymeric network from metal nitrates and citric acid, with ethylene glycol acting as a cross-linker. This chelating approach ensures molecular-level mixing and the homogeneous distribution of multiple cations. Upon the thermal treatment, the organic matrix decomposes, leaving behind a uniform oxide powder. This method is extensively used for complex oxides such as La_1–x_Sr_x_MnO_3_ and LaFeO_3_, where precise stoichiometric control is critical for achieving optimal magnetotransport and magnetic properties. The Pechini method facilitates low-temperature synthesis and a high phase purity, which are essential for the controlled tuning of the colossal magnetoresistance behavior [115,116].

Sol–gel auto-combustion: Auto-combustion techniques involve the use of metal nitrates and organic fuels such as glycine, urea, or citric acid. The exothermic redox reaction between the nitrate and fuel leads to a self-sustained combustion process, rapidly forming fine oxide powders. This method is particularly efficient for the synthesis of ferrites such as NiFe_2_O_4_, CoFe_2_O_4_, and BiFeO_3_. The rapid reaction reduces agglomeration and allows them to produce nanosized particles with high surface areas. These characteristics are beneficial for magnetic applications requiring strong anisotropy or exchange bias effects [117,118].

**Figure 4:**
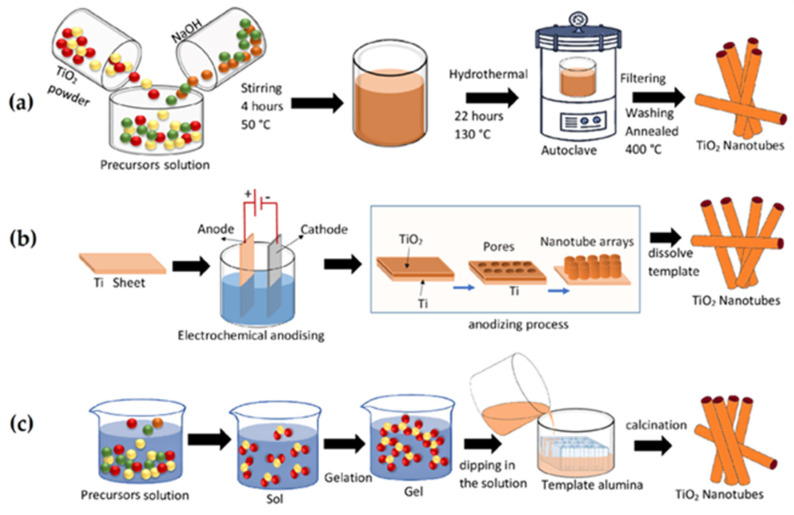
A schematic representation of the main production methods of TiO_2_ nanotubes. (**a**) The hydrothermal method. (**b**) The self-assembled electrochemical anodizing method. (**c**) The sol–gel method [120].

Template-assisted sol–gel: Template-directed sol–gel synthesis uses soft templates (surfactants) or hard templates (porous silica, polystyrene beads) to control the morphology of the final oxide. This approach is valuable for creating mesoporous and hollow structures, such as mesoporous CoFe_2_O_4_ or hollow Fe_3_O_4_ spheres. The confined growth environment provided by the template can influence the grain orientation, magnetic anisotropy, and surface defect density, all of which impact the spintronic functionality. After the gel formation, the template is removed by calcination, yielding a highly ordered magnetic nanostructure [119]. Figure 4 illustrates the main production methods of TiO_2_ nanotubes: the hydrothermal method, the self-assembled electrochemical anodizing method, and the sol–gel method [120].

### 2.4. Examples of Sol–Gel Derived Magnetic Oxide Systems

#### 2.4.1. Co-Doped ZnO (Zn_1−x_Co_x_O)

Zinc oxide is a wide-bandgap semiconductor (~3.3 eV) that becomes magnetically active upon doping with transition metals such as cobalt. The introduction of Co into the ZnO lattice creates localized magnetic moments that can interact via mechanisms such as bound magnetic polarons or through the mediation of oxygen vacancies [121]. These defective structures can lead to room-temperature ferromagnetism, making Co-doped ZnO a promising dilute magnetic semiconductor. Sol–gel synthesis enables fine control over the dopant concentration, crystallite size, and phase purity [122]. Typical Zn_1-x_Co_x_O nanoparticles synthesized via the sol–gel range from 10 to 20 nm in diameter. Their magnetic properties are sensitive to synthesis parameters such as the pH, calcination temperature, and atmosphere, all of which influence the oxygen vacancy formation [123].

**Figure 5:**
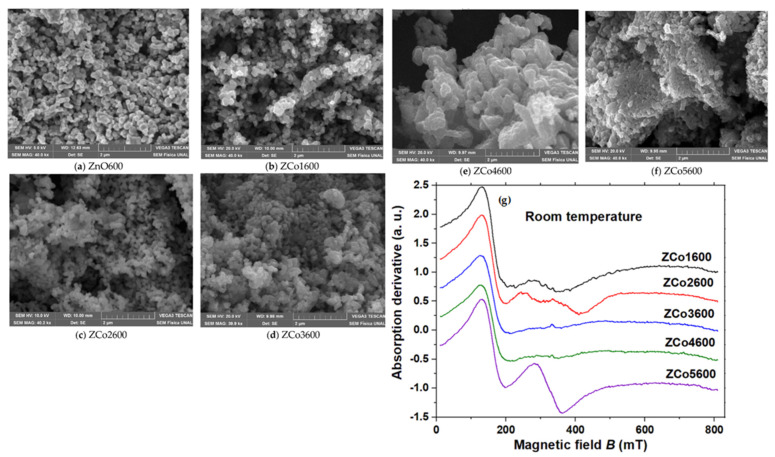
(**a**) SEM micrograph of pure ZnO; (**b**–**f**) SEM micrographs of Co-doped ZnO nanocrystals ZCoX600, where X = 1, 2, 3, 4, and 5, respectively. All samples exhibit spherical morphology with particle agglomeration and porosity due to calcination at 600 °C. (**g**) X-band electron paramagnetic resonance spectra of ZCoX600 samples (X = 1 to 5), recorded at room temperature (295 K) with microwave frequency of 9.44 GHz, showing broadened and structureless features indicative of Co^2+^ paramagnetic centers [124].

Manuel Fernando et al. [124] investigated the structural, magnetic, optical, and photocatalytic properties of Co-doped ZnO nanocrystals synthesized via a sol–gel method. The cobalt dopant introduced various crystal defects, such as zinc and oxygen vacancies, interstitials, antisite defects, and charged oxygen vacancies, enhancing the material’s electronic and photocatalytic behavior. A structural analysis confirmed that Co was successfully incorporated into Zn sites with a deviation of less than 5% between the nominal and actual composition. As shown in Figure 5, scanning electron microscopy revealed a spherical particle morphology with evident porosity and agglomeration due to the calcination at 600 °C and the evolution of gases during synthesis. The magnetic characterization using X-band electron paramagnetic resonance, displayed in Figure 5, identified four distinct Co^2+^ paramagnetic centers, with some demonstrating ferromagnetic coupling. The EPR spectra showed broad, structureless lines, with the previous literature indicating linewidth increases with temperature due to spin–lattice relaxation effects above 60 K, which remained constant below 60 K, suggesting a magnetic origin. No spectral simulations were performed in this work. Photocatalytic experiments on Congo red dye degradation revealed that Co-doped ZnO significantly enhanced the photocatalytic efficiency, achieving over a 50% higher degradation compared to undoped ZnO nanocrystals, highlighting their promise for environmental remediation.

#### 2.4.2. La_1−x_Sr_x_MnO_3_ (LSMO)

LSMO is a mixed-valence perovskite oxide well-known for exhibiting colossal magnetoresistance and a nearly 100% spin polarization at low temperatures [125]. The replacement of La^3+^ with Sr^2+^ introduces Mn^3+^/Mn^4+^ redox couples that mediate double-exchange interactions critical for magnetotransport. The Pechini sol–gel method is ideal for synthesizing this compound, offering excellent control over the Sr content and homogeneity. The phase purity and crystalline quality of the resulting powders or films significantly influence the onset temperature of the metal–insulator transition and the magnitude of the magnetoresistance. Moreover, the grain boundaries and crystallite size, which are tunable via sol–gel parameters, affect the electron transport pathways and spin scattering [126].

**Figure 6:**
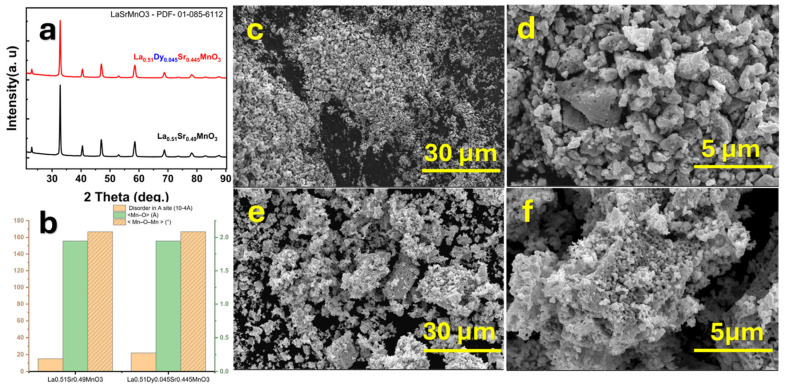
(**a**) X-ray diffraction (XRD) patterns of La_0.51_Sr _0.49_MnO_3_ and Dy-doped La0.51Dy0.045Sr0.445MnO3, showing phase identification using Cu Kα radiation (λ = 1.5406 Å). (**b**) Schematic representation illustrating changes in lattice disorder and Mn–O–Mn bond angles. (**c**–**f**) SEM images of nanoparticle morphology taken at 15 kV, showing porosity and grain size evolution with Dy doping [127].

Smari et al. [127] conducted a comprehensive study on La_0.51_Sr_0.49_MnO_3_ and its Dy-doped counterpart, La_0.51_Dy_0.045_Sr_0.445_MnO_3_, to explore their potential in magnetic hyperthermia applications. As shown in Figure 6, the XRD analysis confirmed that both compositions adopt an orthorhombic perovskite structure, with the Dy incorporation causing noticeable lattice distortion due to the smaller ionic radius of Dy^3+^. These structural changes influence the Mn–O–Mn bond angles and electronic interactions. SEM micrographs revealed a more compact and homogeneously distributed grain morphology in Dy-doped samples, attributed to the strain-induced grain refinement during synthesis. This improved uniformity and reduced grain size visible in the higher magnification images suggest enhancements in the surface area and intergranular connectivity, which are both important for energy dissipation.

**Figure 7:**
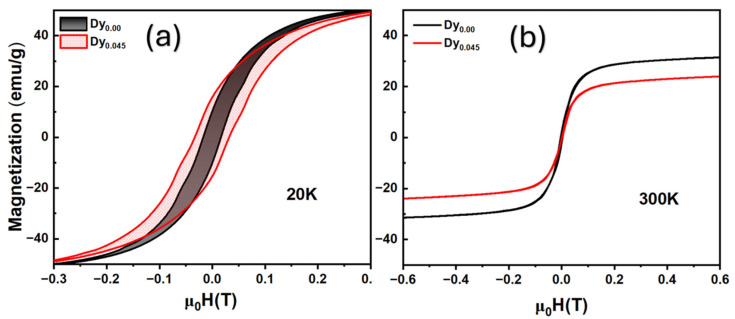
(**a**,**b**) Field-dependent magnetization at 20 K (**a**) and 300 K (**b**) for LSMO and Dy-doped LSMO nanoparticles [127].

The magnetic heating performance, evaluated under alternating magnetic fields, is presented in Figure 7. The Dy-doped samples demonstrated lower SAR values compared to undoped ones, which the authors linked to an enhanced magnetic anisotropy that altered relaxation dynamics. Moreover, the absence of hysteresis in magnetization curves confirms the superparamagnetic nature of both nanoparticle types. The power-law fitting of the SAR versus the magnetic field amplitude showed exponents below two, further supporting the superparamagnetic behavior. This study emphasizes the role of compositional tuning in optimizing the magnetic and heating properties of perovskite-based nanoparticles for biomedical applications.

#### 2.4.3. Fe_3_O_4_ (Magnetite) Nanoparticles

**Figure 8:**
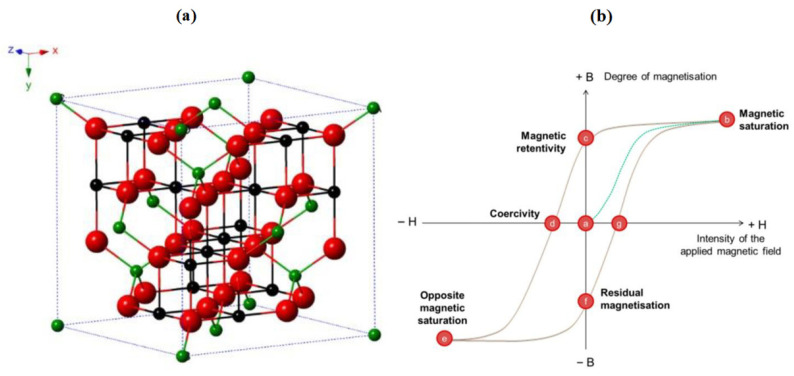
(**a**) The crystal structure of Fe_3_O_4_, showing the inverse spinel arrangement with Fe^2+^ and Fe^3+^ ions in octahedral and tetrahedral sites; (**b**) the hysteresis loop of magnetite nanoparticles, highlighting the ferrimagnetic behavior and their transition to superparamagnetism at the nanoscale [130].

Magnetite is a ferrimagnetic oxide with a high Curie temperature (~850 K) and well-established applications in spintronics and biomedical devices [128]. Sol–gel synthesis allows the production of uniform Fe_3_O_4_ nanoparticles with a controlled size and magnetic characteristics. These nanoparticles often exhibit high saturation magnetization and low coercivity, making them suitable for spin valve structures, magnetic hyperthermia, and spin-based biosensing. The choice of the iron precursor, pH, and calcination profile determines whether the product is Fe_3_O_4_ or its oxidized form γ-Fe_2_O_3_ [129]. Ganapathe et al. [130] presented a detailed overview of magnetite (Fe_3_O_4_) nanoparticles, highlighting their unique structural and magnetic properties that support widespread biomedical applications. As depicted in Figure 8a, magnetite exhibits an inverse spinel crystal structure, with Fe^2+^ and Fe^3+^ ions occupying octahedral sites and Fe^3+^ also populating tetrahedral positions. This configuration, combined with the dynamic electron hopping between Fe^2+^ and Fe^3+^ at octahedral sites, contributes to the magnetite’s electrical conductivity and stability. The spinel unit cell, face-centered along the direction, also determines its fundamental physical traits, such as color changes with oxidation from jet-black magnetite to brownish maghemite (γ-Fe_2_O_3_) and eventually to reddish hematite (α-Fe_2_O_3_) upon annealing.

Magnetically, Fe_3_O_4_ nanoparticles display ferrimagnetism, driven by the antiparallel alignment of Fe^3+^ spins in tetrahedral sites against the parallel orientation of Fe^2+^/Fe^3+^ spins in octahedral sites. This asymmetry generates a net magnetic moment, as illustrated in Figure 8b, where the characteristic hysteresis loop confirms ferrimagnetic behavior at room temperature. However, as the particle size decreases, thermal energy enables a magnetic moment reorientation, transitioning the material into a superparamagnetic regime. The transition temperature, at which this occurs, is typically around 858 K but decreases with the enhanced surface spin contributions and dipolar anisotropy in nanoscale systems. Additionally, the oxygen deficiency at the nanoscale increases the proportion of Fe^2+^ ions, strengthening the magnetic response and increasing the saturation magnetization (Ms). For magnetite nanoparticles, the Ms can reach up to 88.1 emu/g under a 1.5 T field. This high magnetic responsiveness is critical for medical use, enhancing their performance as drug carriers guided by external magnetic fields and as effective contrast agents in MRI. Together, the structural fidelity and tailored magnetic behavior of Fe_3_O_4_ nanoparticles support their strong potential in biomedical nanotechnology.

#### 2.4.4. NiFe_2_O_4_ and CoFe_2_O_4_ Ferrites

**Figure 9:**
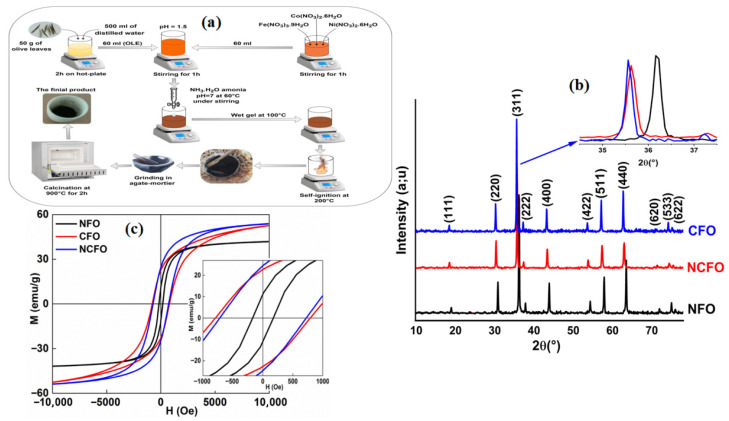
(**a**) A schematic representation of the green sol–gel auto-combustion synthesis process of NiFe_2_O_4_, CoFe_2_O_4_, and Ni_0.5_Co_0.5_Fe_2_O_4_ nanoparticles using olive leaf extract (OLE) as fuel. (**b**) XRD patterns confirming the single-phase cubic spinel structure of NFO, CFO, and NCFO ferrites, with (311) principal peaks shown in the inset. (**c**) Room-temperature M–H hysteresis loops of the synthesized ferrite samples, illustrating their magnetic behavior [134].

These ferrites possess an inverse spinel structure and exhibit significant magnetic anisotropy, which is advantageous for high-frequency spintronic applications. NiFe_2_O_4_ is a soft magnetic material with moderate coercivity, while CoFe_2_O_4_ has a high coercivity and magnetocrystalline anisotropy. Sol–gel auto-combustion and citrate–nitrate routes are commonly used to prepare these ferrites with a fine particle size and phase purity [131]. The magnetic properties of these materials can be tuned by adjusting the synthesis route, cation distribution, and thermal treatment conditions. Surface effects, particularly in nanoparticles, contribute to the exchange bias and coercivity enhancement [132,133]. Boussafel et al. [134] explored a sustainable route to synthesize ferrite nanoparticles, specifically NiFe_2_O_4_ (NFO), CoFe_2_O_4_ (CFO), and Ni_0.5_Co_0.5_Fe_2_O_4_ (NCFO), using a sol–gel auto-combustion technique where olive leaf extract (OLE) served as a natural fuel. This green synthesis method aimed to minimize the environmental impact while maintaining effective control over the particle size, shape, and magnetic performance. The procedure, summarized in Figure 9a, involved preparing the OLE from boiled olive leaves, combining it with metal nitrate solutions, and initiating a self-sustaining combustion reaction that produced porous powders, later calcined at 900 °C. The structural analysis using XRD, shown in Figure 9b, confirmed that all samples crystallized into a single-phase cubic spinel structure (space group Fd-3m), with primary diffraction peaks indicating high crystallinity. The shift in 2θ values, especially for NCFO and NFO, was attributed to the lattice contraction caused by the substitution of cobalt ions with smaller nickel ions. The Rietveld refinement further confirmed these structural adjustments, including variations in the lattice parameters, crystallite size, and oxygen position. The study also examined the magnetic behavior at room temperature. As shown in Figure 9c, magnetization hysteresis loops indicated soft magnetic properties for all three ferrites, with NFO displaying a narrow, symmetric loop, while CFO and NCFO exhibited an enhanced coercivity and saturation magnetization. These magnetic differences were linked to the cation distribution within tetrahedral and octahedral lattice sites, as well as the influence of the crystal structure, size, and degree of inversion.

#### 2.4.5. Mn-Doped TiO_2_

**Figure 10:**
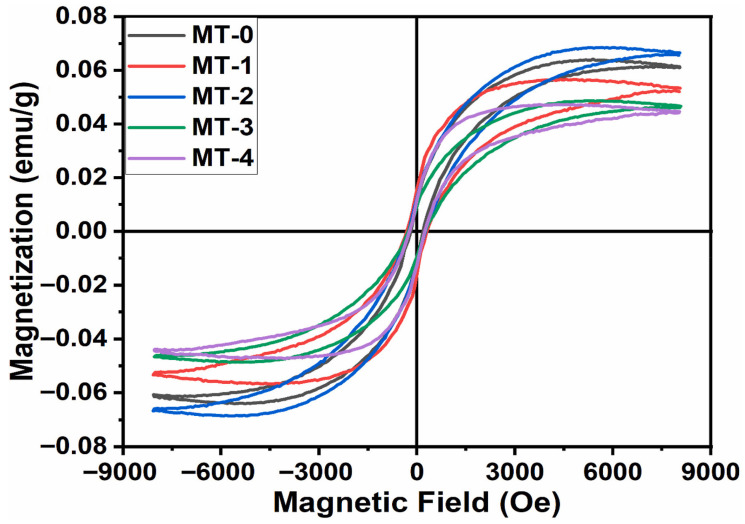
Magnetization at room temperature against magnetic field of pure and Mg-doped TiO_2_ nanoparticles [136].

Titanium dioxide, in both anatase and rutile forms, becomes magnetically active when doped with Mn. The magnetic behavior is typically attributed to the defect-mediated exchange involving oxygen vacancies and Mn^2+^/Mn^3+^ redox centers. The sol–gel method enables a uniform Mn incorporation and precise phase control, which are essential for achieving consistent magnetic properties. Mn-doped TiO_2_ is studied for use in spin-filtering devices and photocatalysis with a magnetic recovery capability [135]. Adedokun et al. [136] reported the synthesis of Mg-doped anatase TiO_2_ nanoparticles using a sol–gel technique, targeting their application in spintronic and optoelectronic devices. The structural characterization using XRD confirmed the retention of the anatase tetragonal phase across all Mg doping levels (x = 0.00–0.04), without the emergence of secondary phases, implying a successful Mg integration into the TiO_2_ lattice. Optical measurements via UV–Vis–NIR diffuse reflectance spectroscopy showed a slight band gap widening from 3.25 eV (undoped) to 3.33 eV (4% Mg-doped), attributed to a blue shift in the absorption edge. The photoluminescence analysis further revealed that the Mg doping effectively reduced the recombination rate of photo-generated electron–hole pairs, enhancing the charge carrier separation. Magnetic properties studied through vibrating sample magnetometry at room temperature demonstrated soft ferromagnetic behavior in both undoped and Mg-doped samples. Figure 10 illustrates the M–H hysteresis loops, where all compositions show clear room-temperature ferromagnetism, with non-zero coercivity (206–309 Oe) and remanent magnetization. The 2% Mg-doped sample exhibited the highest saturation magnetization. These results underline the material’s potential for future applications in magneto-optical and spintronic technologies.

#### 2.4.6. CeO_2_ Doped with Fe or Co

**Figure 11:**
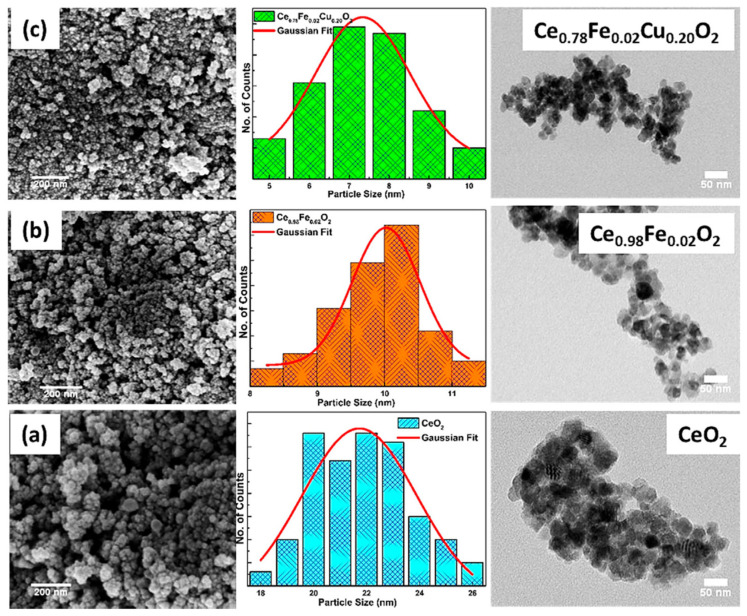
FE-SEM micrographs of (**a**) pure CeO_2_, (**b**) Ce_0.98_Fe_0.02_O_2_, and (**c**) Ce_0.78_Fe_0.02_Cu_0.20_O_2_ nanoparticles, along with the corresponding TEM images [139].

Cerium dioxide is a fluorite-structured oxide that is normally non-magnetic. However, doping with Fe or Co induces ferromagnetism through defect engineering. The Ce^3+^/Ce^4+^ redox couple facilitates charge compensation and oxygen vacancy formation, which are crucial for magnetic ordering. Sol–gel synthesis allows for the precise control of the dopant level, crystallite size, and Ce oxidation state ratio. Such materials are being explored for multifunctional spintronic devices that also exhibit catalytic or redox activity [137,138]. Shalendra et al. [139] investigated the influence of Fe and Cu co-doping on the structural, magnetic, and electrochemical properties of CeO_2_ nanoparticles synthesized through a co-precipitation technique. X-ray diffraction results confirmed the formation of a single-phase cubic fluorite structure across all samples without any secondary impurities. Doping with Fe and Cu led to a reduction in the crystallite size, with the co-doped Ce_0.78_Fe_0.02_Cu_0.20_O_2_ nanoparticles exhibiting the smallest average size of 5.59 nm. The morphology, analyzed using FE-SEM and TEM, revealed spherical particles with slight agglomeration, as presented in Figure 11, which displays the surface and internal structure of pure, Fe-doped, and co-doped samples.

**Figure 12:**
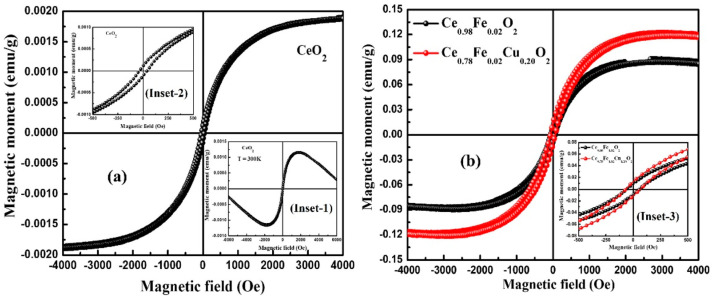
(**a**,**b**) M-H plots of CeO_2_, Ce_0.98_Fe_0.02_O_2_, and Ce_0.78_Fe_0.02_Cu_0.20_O_2_ nanoparticles. Insect-1 shows the M-H curve of pure CeO_2_ nanoparticles along with the diamagnetic contribution in the field range of ±6000 Oe. Inset-2 represents the M-H curve of CeO_2_ nanoparticles in the low-field range (±500 Oe). Inset-3 represents the M-H curve of Ce_0.98_Fe _0.02_ O_2_ and Ce_0.78_Fe_0.02_Cu_0.20_O_2_ nanoparticles in the low-field range (±500 Oe) [139].

The Raman spectra indicated defect-related shifts, suggesting the presence of oxygen vacancies associated with doping. The UV–visible analysis showed a lowered band gap of around 1.7 eV, which may benefit the optoelectronic performance. Magnetic measurements at room temperature, illustrated in Figure 12, revealed the weak ferromagnetic behavior in undoped CeO_2_, attributed to intrinsic oxygen vacancies, but a clear enhancement in magnetization with the Fe and Cu co-doping. The saturation magnetization increased from 9.0 × 10^−2^ to 12.2 × 10^−2^ emu/g, and the remanent magnetization and coercive field also rose. This improved magnetic behavior is interpreted via the F-center exchange mechanism, where electrons trapped in oxygen vacancies mediate ferromagnetic coupling between transition metal ions.

#### 2.4.7. Mechanistic Insights and Critical Perspectives

While sol–gel synthesis enables the formation of magnetic oxide nanostructures with controlled sizes and doping, the underlying mechanisms responsible for their magnetic behavior are complex and still debated. Among the most studied mechanisms is the bound magnetic polaron model, particularly relevant in ZnO- and TiO_2_-based dilute magnetic semiconductors. In this model, oxygen vacancies trap charge carriers that locally polarize surrounding magnetic dopants (e.g., Co^2+^ or Mn^2+^), forming magnetically active polarons. The overlap of these polarons leads to long-range ferromagnetic ordering. Another key mechanism is the F-center exchange, where electrons localized in oxygen vacancies (F-centers) mediate indirect exchange interactions between dopant ions, especially in fluorite-type structures like CeO_2_. In perovskite oxides such as La_1−x_Sr_x_MnO_3_, the double-exchange mechanism dominates the itinerant electron hop between Mn^3+^ and Mn^4+^ ions, aligning their spins through kinetic energy gains. Despite the diverse mechanisms proposed, it remains challenging to distinguish intrinsic magnetic effects from those arising due to secondary phases or dopant clustering, which may inadvertently form during sol–gel processing.

Therefore, a rigorous correlation between magnetic properties and structural/microstructural characterization, such as high-resolution TEM, XPS, Mössbauer spectroscopy, and EPR, is essential. Furthermore, inconsistent reports in the literature regarding Curie temperatures and saturation magnetizations of similar systems highlight a lack of reproducible processing protocols. Going forward, sol–gel chemistry must evolve toward better defect engineering, controlled doping strategies, and integration with machine learning tools to close the gap between empirical observations and theoretical models. To synthesize the insights from various oxide systems discussed above, in Table 3 we present a comparative overview of representative sol–gel-synthesized metal oxides, highlighting their synthesis approaches, doping strategies, key magnetic behaviors, and relevance to spintronic applications. This comparison is intended to assist researchers in identifying the optimal materials and synthesis routes for specific functional requirements.

### 2.5. Processing Parameters and Their Effects

The effectiveness of sol–gel synthesis in tailoring spintronic properties is strongly influenced by several key processing parameters. These include the calcination temperature, dopant concentration, pH of the precursor solution, atmospheric conditions during heat treatment, and aging duration of the gel. Each of these parameters plays a critical role in defining the final crystalline, morphology, phase purity, defect structure, and magnetic behavior of the synthesized nanomaterials. The calcination temperature directly affects the crystallite size, phase formation, and defect density. For example, increasing the temperature can enhance the crystallinity and reduce defects but may also lead to dopant segregation or secondary phase formation. In Co-doped ZnO or Mn-doped TiO_2_, insufficient calcination may result in amorphous or poorly crystalline phases with diminished magnetic responses, whereas an excessive heat treatment can lead to the formation of unwanted metallic clusters [140].

The dopant concentration must be carefully optimized to achieve the desired magnetic behavior. While low concentrations may not induce sufficient magnetic coupling, high dopant levels can lead to clustering, secondary phases, or antiferromagnetic interactions. For instance, in LSMO or CoFe_2_O_4_ systems, the magnetic saturation and coercivity are highly sensitive to cation ratios [141]. The pH of the sol impacts the hydrolysis and condensation rates, which in turn affect nanoparticle nucleation and growth. Acidic conditions often favor the formation of more uniform and smaller particles, while basic environments can accelerate condensation, sometimes at the expense of homogeneity. This is particularly important in the synthesis of TiO_2_- and CeO_2_-based DMSs [142]. Atmospheric conditions during drying and calcination significantly influence the oxygen vacancy concentration and oxidation states. Processing in a reducing or inert atmosphere can stabilize lower oxidation states (e.g., Fe^2+^, Ce^3+^), which are often linked to enhanced ferromagnetism. Conversely, calcination in oxygen-rich atmospheres can suppress the formation of oxygen vacancies, thereby reducing defect-mediated ferromagnetism that is typically attributed to mechanisms such as F-center exchange or bound magnetic polarons in oxide systems like ZnO and TiO_2_ [143].

The aging time allows the gel network to strengthen and evolve before drying. Extended aging can improve particle connectivity and structural ordering, leading to more stable phases and better magnetic coupling [144]. A concise summary of these relationships is provided in Table 4. Understanding and precisely tuning these parameters is essential for developing reproducible, high-performance spintronic materials using sol–gel routes. Future studies combining in situ diagnostics and multivariate optimization may further improve the control over these critical synthesis variables. Sol–gel conditions that favor spin polarization typically promote controlled defect formation, homogeneous dopant incorporation, and optimized oxidation states. For example, low-to-moderate calcination temperatures (400–600 °C) help retain oxygen vacancies that facilitate F-center exchange interactions, especially in Co-doped ZnO and Mn-doped TiO_2_. Annealing under reducing or inert atmospheres (e.g., N_2_, Ar/H_2_) stabilizes lower oxidation states such as Fe^2+^ or Mn^3+^, which are critical for double-exchange mechanisms in perovskites like LSMO. Additionally, the use of chelating agents like citric acid improves the dopant distribution and minimizes magnetic inhomogeneity. Acidic sol–gel pH conditions often yield smaller particles and enhance surface spin polarization but may require a careful post-treatment to avoid agglomeration or secondary phases. Overall, tuning these parameters provides a robust pathway to tailor the spin polarization in oxide nanostructures.

A critical examination of sol–gel studies reveals notable differences in structural and magnetic outcomes depending on synthesis routes. For example, Co-doped ZnO prepared via an alkoxide-based sol–gel often exhibits room-temperature ferromagnetism that is sensitive to oxygen vacancy concentrations, while LSMO synthesized via the Pechini method shows enhanced magnetoresistive behavior due to superior dopant homogeneity and lattice distortion control. Similarly, Fe_3_O_4_ nanoparticles synthesized using auto-combustion routes demonstrate high saturation magnetization but may suffer from agglomeration and phase impurities unless optimized carefully. The choice of the metal dopant has a profound effect on the resulting magnetic behavior of sol–gel-derived oxides. Transition metals such as Co^2+^, Mn^2+^, Fe^3+^, and Ni^2+^ contribute differently based on their unpaired d-electrons, oxidation state, and crystal field stabilization energy. For instance, Co^2+^ in ZnO induces magnetic ordering via localized moment interactions enhanced by oxygen vacancies. In contrast, Mn^3+^/Mn^4+^ pairs in LSMO engage in double-exchange mechanisms critical for colossal magnetoresistance. Furthermore, an ionic radius mismatch between the dopant and host cations affects the lattice strain and dopant solubility. Rare-earth dopants like Dy^3+^ introduce a lattice distortion and affect the magnetic anisotropy, as seen in Dy–LSMO systems. The preference for octahedral vs. tetrahedral lattice sites also determines whether dopants enhance the ferromagnetism or disrupt long-range ordering. As such, selecting dopants with a compatible ionic size, oxidation state, and magnetic moment is essential for engineering targeted spintronic functionality.

#### Effect of Sol–Gel Parameters on Magnetic Properties

The magnetic behavior of sol–gel-derived nanomaterials is highly sensitive to chemical synthesis parameters. The pH, for instance, controls the hydrolysis and condensation rates of metal precursors. An acidic pH (3–5) often leads to smaller, more uniform particles with a higher surface defect density, which can enhance ferromagnetism in dilute magnetic oxides like Co-doped ZnO. Alkaline conditions (>8), on the other hand, may favor rapid condensation but can result in non-uniform growth or unwanted phase separation. Chelating agents such as citric acid, tartaric acid, and EDTA serve to coordinate metal ions, enabling homogeneous mixing and preventing dopant segregation. In Pechini-type sol–gel methods, citric acid forms stable metal complexes that yield high-purity perovskites (e.g., LSMO) with well-dispersed dopants crucial for achieving consistent double-exchange-mediated ferromagnetism. The type of metal precursor (alkoxides vs. nitrates) also influences the final magnetic outcome. Alkoxides typically hydrolyze rapidly and are suitable for single-component oxides (e.g., TiO_2_), while nitrate or acetate salts allow for better control in multicomponent systems and are less sensitive to ambient moisture. The precursor selection impacts oxidation states and ultimately the valence distribution in mixed-valent systems like CeO_2_ or Fe_3_O_4_. Thus, controlling these parameters offers a direct route to engineering magnetic oxide properties for spintronic applications.

### 2.6. Challenges and Considerations

Despite its versatility and advantages, the sol–gel method presents several intrinsic and extrinsic challenges that must be addressed to realize its full potential for spintronic material development. The foremost concern is the formation of secondary phases, particularly metallic or oxide-rich dopant clusters that can dominate the observed magnetism. These extrinsic contributions can obscure intrinsic magnetic behavior and lead to inconsistent or irreproducible results [145,146]. The doping efficiency is another critical factor. Achieving a uniform dopant incorporation at the desired oxidation state is non-trivial, particularly in multivalent systems such as TiO_2_ or CeO_2_. Uncontrolled redox chemistry during sol preparation or calcination can result in a mixture of valence states, which affects both the magnetic coupling and carrier concentration. In systems like Co-doped ZnO or Fe-doped SnO_2_, even slight variations in the dopant environment can shift the material from paramagnetic to ferromagnetic [147,148].

A key challenge in optimizing magnetic oxide nanostructures lies in simultaneously achieving a high crystallinity, a controlled defect distribution, and stable magnetic characteristics. Crystallinity is typically improved through thermal treatment; however, excessive calcination can reduce beneficial defects like oxygen vacancies or cause dopant segregation. For example, while Co–ZnO benefits from oxygen-defect-induced magnetism, high-temperature annealing may anneal out such vacancies, reducing spin polarization. The defect distribution, such as oxygen vacancies or cation interstitials, must be finely tuned. Uncontrolled defect clustering may lead to parasitic magnetic phases or superparamagnetism. In perovskite systems like LSMO, a non-uniform dopant distribution can impair double-exchange interactions, lowering the Curie temperature and magnetoresistance. Similarly, in ferrites, a cationic redistribution during synthesis can shift the material from soft to hard magnetic behavior unpredictably. Finally, magnetic characteristics like coercivity, saturation magnetization, and magnetic anisotropy are highly sensitive to the particle morphology, grain boundaries, and secondary phase formation. These variables are interconnected, and optimizing one property often compromises another. Thus, a multi-variable, iterative optimization approach potentially involving in situ diagnostics and machine learning is necessary for reliable control.

Defect-induced magnetism, while often exploited to achieve RTFM, presents interpretative difficulties. Oxygen vacancies, interstitial cations, and lattice distortions can all induce localized magnetic moments, but their contributions are sensitive to synthesis and measurement conditions. This makes it challenging to establish clear structure–property relationships without advanced spectroscopic or microscopic techniques. Reproducibility and scalability also remain issues for industrial application [149]. Although the sol–gel route is inherently scalable in solutions, translating laboratory recipes to uniform, large-area thin films or device-quality nanostructures is non-trivial. Variations in drying kinetics, shrinkage, and crack formation during thermal treatment can introduce inconsistencies in the film morphology and performance. To address these challenges, several strategies have been proposed and tested.

In situ spectroscopic monitoring (e.g., FTIR, UV–vis, Raman) during sol aging and gelation can help track hydrolysis/condensation dynamics and identify optimal reaction windows. Controlled atmosphere calcination (e.g., N_2_, Ar, or forming gas) enables the precise control over oxygen stoichiometry and dopant oxidation states [150]. Chelating agents (e.g., EDTA, citric acid, tartaric acid) can stabilize metal ions and promote uniform coordination environments, enhancing the dopant dispersion. Multistep annealing or microwave-assisted processing can reduce the energy input and improve the uniformity of nanocrystalline domains. Ultimately, integrating sol–gel synthesis with real-time diagnostics, combinatorial chemistry, and post-synthesis characterization (e.g., XPS, EPR, TEM) will be crucial to overcoming these limitations. By bridging the gap between chemical control and physical performance, these advances can help unlock the full potential of sol–gel-derived nanomaterials in next-generation spintronic devices [151].

Beyond the scientific synthesis challenges, there are major hurdles in transitioning sol–gel-based magnetic oxides from laboratory-scale studies to commercial spintronic devices. One key issue is achieving film uniformity and surface smoothness over large substrate areas, as sol–gel coatings are prone to cracking during drying or calcination. Furthermore, dopant segregation and the formation of metallic clusters can undermine device reproducibility, especially in thin-film tunnel junctions or spin injectors. The compatibility of sol–gel synthesis with semiconductor manufacturing is another concern; temperature limitations, substrate constraints, and contamination control must be carefully addressed for CMOS integration. There is also a significant gap in device-level testing: while materials often exhibit promising magnetic properties at the nanoscale, few studies validate spin injection efficiency, coherence lengths, or device longevity under operational conditions. Tackling these issues will require not only better control over synthesis parameters but also the use of real-time monitoring tools (e.g., in situ spectroscopy, thermal imaging), hybrid integration techniques, and standardization protocols to enable cross-lab reproducibility.

## 3. Recent Advances and Applications in Spintronics

Spintronics, which exploits the spin degree of freedom of electrons along with their charge, has emerged as a frontier in modern electronics. The integration of spintronic principles into materials offers the possibility of developing devices with a higher speed, lower power consumption, and increased data storage density. Metal oxide nanostructures synthesized through sol–gel techniques are of particular interest in this field due to their ease of processing, tunable physical properties, and potential for low-cost, large-scale fabrication. In recent years, there have been notable advancements in the application of sol–gel-derived metal oxides in spintronic devices. These include the development of dilute magnetic oxides, spin-polarized transport systems, multifunctional materials, and novel device architectures [152,153].

### 3.1. Dilute Magnetic Oxides and Room-Temperature Ferromagnetism

One of the critical challenges in spintronics is achieving ferromagnetism at room temperature in semiconducting systems. Dilute magnetic oxides, where a small percentage of transition metal ions (such as Co, Mn, Ni, or Fe) are doped into wide-bandgap oxide semiconductors (like ZnO, TiO_2_, SnO_2_), have shown promise in this area [154,155]. Sol–gel-synthesized DMOs have been reported to exhibit room-temperature ferromagnetism, a key requirement for practical spintronic applications. For instance, Co-doped ZnO thin films prepared by sol–gel spin coating techniques have demonstrated significant magnetic ordering at room temperature [156]. Ferromagnetism is believed to be mediated by a combination of exchange interactions and the presence of defects, particularly oxygen vacancies, which can contribute to long-range magnetic ordering. Similarly, Fe-doped TiO_2_ nanostructures synthesized via sol–gel methods exhibit stable magnetic behavior, attributed to substitutional doping and defect-induced magnetic coupling [157]. In many cases, annealing treatments and atmospheric control during synthesis can significantly affect magnetic properties. Oxygen-deficient environments often enhance ferromagnetism due to increased carrier concentrations. However, the exact origin of ferromagnetism in these systems is still debated, as secondary phase formation or the clustering of dopant atoms may also contribute to the observed magnetism. Therefore, precise control over doping levels and synthesis conditions remains crucial.

**Figure 13:**
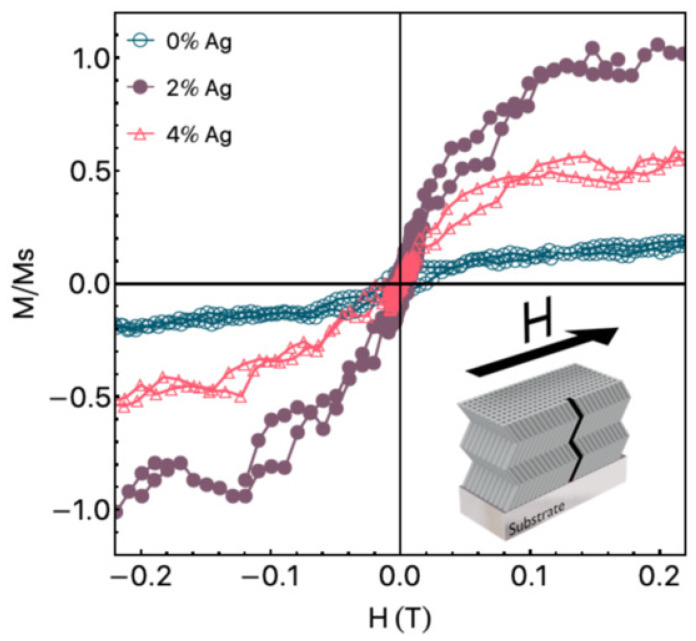
Normalized magnetization curves for the ZnO film and the Ag-doped ZnO films. The magnetization curves are normalized by the saturation magnetization obtained for the ZnO:2Ag film. The inset depicts the field’s applied direction during the magnetization measurements [158].

Correa et al. [158] explored how glancing angle deposition sputtering influences room-temperature ferromagnetism in both pure ZnO and Ag-doped ZnO thin films featuring a distinctive zigzag-like columnar morphology. Magnetic measurements, as illustrated in Figure 13, revealed a clear ferromagnetic response for all samples, even in the undoped ZnO films, contrary to many earlier studies that reported no such behavior. By removing the background diamagnetic signals, the authors could isolate the intrinsic magnetic contributions of the ZnO matrix. The films exhibited in-plane isotropic hysteresis loops, with coercive fields of 16.4 mT, 5.1 mT, and 2.2 mT for the pure ZnO, ZnO:2Ag, and ZnO:4Ag samples, respectively. The saturation magnetization, normalized to the ZnO:2Ag film, reached as high as 0.78 emu/cm^3^ in the undoped case. This enhancement in magnetization was attributed to the presence of Zn vacancies induced by the sputtering process and the morphological disorder introduced by the columnar growth structure, both of which generate localized magnetic moments. Interestingly, introducing 2% Ag significantly boosted the magnetic moment, while further increasing the Ag to 4% caused a decline, suggesting an optimal doping range for maximizing ferromagnetic ordering. This non-monotonic trend parallels previous findings in other non-ferromagnetic metal-doped systems like Cu-doped ZnO. The study highlights a promising route for tailoring multifunctional ZnO-based films, where magnetism can complement electrical functionality, potentially benefitting advanced biosensor platforms.

### 3.2. Spin-Dependent Transport and Magnetoresistance

Sol–gel-derived metal oxide nanostructures have been actively investigated for their spin transport properties, particularly in the context of magnetoresistive devices such as spin valves and MTJs. These devices rely on the manipulation of spin-polarized currents and the ability of the material to maintain spin coherence and polarization over extended distances. Materials like Fe_3_O_4_, known for high spin polarization and a high Curie temperature, have been synthesized via sol–gel auto-combustion methods and incorporated into spintronic device architectures [159]. Thin films and nanoparticles of Fe_3_O_4_ produced by sol–gel processes have shown promising magnetoresistance effects when integrated with insulating oxide barriers such as MgO or Al_2_O_3_ [160]. LSMO, a well-known half-metallic ferromagnet, has also been successfully prepared by sol–gel techniques. When used as an electrode in MTJs, LSMO films show high spin polarization and low resistivity, making them ideal for spin injection applications. The sol–gel route enables good control over the microstructure and interface sharpness, which are critical for achieving high tunneling magnetoresistance [161].

**Figure 14:**
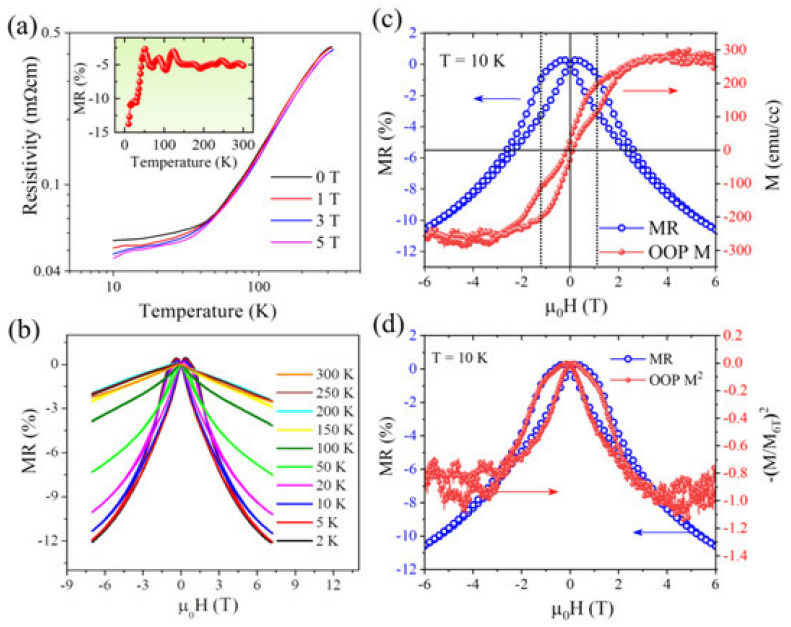
Magnetotransport characteristics of MnP thin films under varying temperature and magnetic field conditions: (**a**) Resistivity as a function of temperature (10–305 K) under different magnetic fields, with the inset showing magnetoresistance behavior at 5 T; (**b**) magnetic field-dependent magnetoresistance across multiple temperatures, with the inset highlighting the MR response at 2 K and indicating the sweep direction; (**c**) a comparative plot of the magnetoresistance and out-of-plane magnetization at 10 K; (**d**) the correlation between the magnetoresistance and squared reduced magnetization at 10 K, demonstrating the influence of intergranular spin-dependent tunneling [162].

Muchharla et al. [162] explored the magnetotransport behavior of highly crystalline MnP nanorod thin films grown via molecular beam epitaxy, focusing on the interplay between the magnetic ordering and electrical resistivity across a wide temperature range. Their results, summarized in Figure 14, reveal a pronounced negative magnetoresistance (MR) effect that emerges below 50 K, a temperature region associated with the formation of a stable helical magnetic phase. In Figure 14a, temperature-dependent resistivity curves recorded under various magnetic fields exhibit metallic behavior, and the inset clearly shows a strong enhancement of the MR below 50 K. The further investigation in Figure 14b, where the magnetic field-dependent MR is measured across different temperatures, demonstrates a butterfly-shaped hysteresis profile at low temperatures, particularly at 2 K, confirming the field-sensitive nature of magnetic ordering. This hysteresis diminishes significantly above 50 K and disappears near 100 K, coinciding with the FM to HM transition. The correlation between the MR and magnetization is made more explicit in Figure 14c, which compares the MR isotherm and out-of-plane magnetization loop at 10 K both displaying aligned switching features near the coercive field. Finally, Figure 14d plots the MR as a function of the squared reduced magnetization, confirming that the MR closely follows the magnetization trend, as predicted by models of spin-dependent intergranular tunneling. These results collectively emphasize that the dominant MR mechanism in MnP nanorod films is rooted in the spin-polarized tunneling across grain boundaries, enabled by the nanostructured geometry and magnetic texture of the material. Such behavior positions MnP films as strong candidates for low-temperature spintronic devices, where magnetic phase transitions can be utilized to tune electronic transport.

### 3.3. Multifunctional Nanostructures for Spintronics

In addition to pure magnetic and spin transport behavior, sol–gel-synthesized nanostructures offer multifunctional properties that are valuable for advanced spintronic applications. Materials that exhibit a combination of magnetic, semiconducting, and optical behavior are particularly useful for multifunctional devices such as spin-light-emitting diodes, spin-photo detectors, and spin transistors [163]. Mn-doped ZnO nanostructures prepared via sol–gel have shown both photoluminescence and magnetic ordering, indicating the potential to couple optical transitions with spin dynamics. Similarly, Ni-doped SnO_2_ films synthesized through sol–gel methods exhibit magnetism and tunable electrical conductivity, enabling their use in devices where magnetic and electronic responses are coupled [164]. Furthermore, the ability to engineer materials that respond to external electric or magnetic fields, such as field-controlled magnetic semiconductors, opens new pathways for developing spin field-effect transistors and magnetoelectric memory devices. For conventional spintronic functions, such as spin injection, spin filtering, and magnetoresistance, sol–gel-synthesized magnetic oxides are also gaining attention for their bio-compatible magnetism. This emerging field involves the integration of magnetic nanostructures such as ferrites or Fe_3_O_4_ with biomedical applications including magnetic hyperthermia, MRI contrast enhancement, and targeted drug delivery. The compatibility of sol–gel synthesis with bio-compatible substrates and its ability to produce nanoparticles with tunable magnetic properties makes it particularly suitable for such multifunctional applications.

In contrast to earlier studies that focused primarily on metallic spintronic systems, recent efforts have shifted toward the integration of sol–gel-derived metal oxides due to their compatibility with CMOS platforms, low processing temperatures, and environmental sustainability. Co-doped ZnO thin films fabricated via spin-coating have shown a promising performance in spin light-emitting diodes and spin field-effect transistors, exhibiting a stable room-temperature magnetism along with tunable optical emissions. Magnetite (Fe_3_O_4_) nanoparticles produced through sol–gel auto-combustion have been successfully implemented in magnetic tunnel junctions, where their high Curie temperature and strong spin polarization enhance the spin filtering efficiency. Similarly, La_1−x_Sr_x_MnO_3_ thin films synthesized by sol–gel techniques have been incorporated into spin valves and magnetoresistive sensors, delivering high signal sensitivity and robust magnetoresistive effects. NiFe_2_O_4_ powders derived from sol–gel routes have found use in high-frequency RF applications, where their soft magnetic character and tunable coercivity contribute to low-loss magnetic components. Beyond spin transport devices, sol–gel-processed oxides are also under investigation for emerging applications in spin caloritronics, magneto-optical modulation, and magnetoelectric memory, underscoring their multifunctional potential in next-generation spintronic systems.

### 3.4. Nanostructure Morphology and Dimensional Effects

The sol–gel process allows for the precise tuning of the nanostructure morphology, which plays a critical role in determining the spintronic performance [165]. By adjusting synthesis parameters, such as the pH, precursor concentration, and annealing temperature, it is possible to produce nanostructures in the form of thin films, nanoparticles, nanorods, or porous aerogels. One-dimensional nanostructures like Co-doped ZnO nanorods exhibit enhanced surface areas and quantum confinement effects, which can amplify surface spin polarization and improve the magnetic response. Similarly, porous SnO_2_ aerogels doped with transition metals show superparamagnetic behavior due to the high density of surface spins and grain boundaries [166]. These morphological features significantly affect the spin transport, as surface defects, grain boundaries, and quantum confinement influence both magnetic interactions and electron scattering processes. Tailoring the structure at the nanoscale allows for the optimization of the spin lifetime, spin diffusion length, and magnetic anisotropy key parameters for efficient spintronic functionality.

## 4. Outlook and Future Directions

As spintronics evolves from fundamental magnetotransport studies to real-world applications in data storage, quantum computing, and energy efficient electronics, the role of sol–gel synthesis is set to become even more central. The unique advantages of the sol–gel method, including its molecular-level control over compositions, its low-temperature processing, and its ability to produce diverse nanostructures, make it exceptionally well-suited for addressing the next-generation challenges in spintronic material development. One of the most promising frontiers lies in defect engineering at the atomic scale. The sol–gel approach enables the fine-tuning of defect landscapes such as oxygen vacancies, cation interstitials, and grain boundary chemistry features that critically influence ferromagnetism and spin transport. Coupling sol–gel synthesis with advanced spectroscopic techniques, like X-ray photoelectron spectroscopy, extended X-ray absorption fine structure, and in situ Raman/FTIR, can provide real-time insight into the evolution of these defects. Such data-driven feedback loops could allow unprecedented control over magnetic ordering and dopant activation.

Another exciting direction is the integration of sol–gel-derived oxides with emerging 2D and hybrid materials. By combining magnetic metal oxides with layered materials like graphene, transition metal dichalcogenides, MXenes, or even organic semiconductors, researchers can design novel heterostructures that exhibit synergistic properties such as spin filtering, gate-tunable magnetism, or room-temperature spin–orbit torque effects. Sol–gel’s ability to produce conformal, uniform coatings at low temperatures is particularly advantageous for these fragile, substrate-sensitive systems. Such interfaces may enable flexible or wearable spintronic devices and pave the way for multifunctional bio-integrated platforms. In response to growing environmental concerns, there is also a push toward green sol–gel chemistry. This includes the development of water-based systems that replace hazardous organic solvents, as well as the use of renewable or biodegradable templates and chelating agents. Incorporating green chemistry principles into sol–gel synthesis not only aligns with sustainability goals but also enhances biocompatibility for applications in biospintronics and medical nanotechnology.

In parallel, the rise of spin-caloritronic and thermoelectric applications is driving the demand for magnetic materials that respond sensitively to thermal gradients. Sol–gel synthesis, with its capacity to engineer both microstructure and defect landscapes, presents a versatile platform for optimizing materials for the spin Seebeck effect, the anomalous Nernst effect, and other thermomagnetic phenomena. Such materials could enable energy-efficient spin-based logic, waste heat harvesting, or temperature-sensitive spin sensors. Furthermore, the integration of artificial intelligence and machine learning (ML) into sol–gel materials research is poised to accelerate the discovery and optimization of spintronic nanostructures. High-throughput synthesis and characterization, coupled with predictive modeling, can drastically reduce the trial-and-error typically involved in materials development. By leveraging sol–gel’s tunability with AI-guided design and robotic synthesis, the field moves closer to autonomous materials discovery, positioning sol–gel-synthesized nanostructures as central components in next-generation multifunctional spintronic devices.

Although sol–gel synthesis offers significant promise, several critical knowledge gaps persist. First, the precise role of oxygen vacancies and their interplay with dopant ions remains poorly understood across different oxide systems. For example, contradictory magnetic trends have been reported for identically doped ZnO and TiO_2_ samples, highlighting the sensitivity of spin behavior to subtle changes in processing. Second, while structural control is often achieved, electronic and magnetic property tuning remains empirical. There is a strong need for predictive models that link sol–gel chemistry with spintronic functionality. Third, the integration of sol–gel-derived oxides with 2D materials (e.g., graphene, MoS_2_) or organic semiconductors remains largely unexplored but could unlock novel hybrid spintronic architectures. Lastly, scalable, eco-friendly sol–gel routes using aqueous precursors and biodegradable templates are essential for future bio-integrated or wearable spintronic systems. Addressing these research priorities will require a combination of materials informatics, real-time diagnostics, and interdisciplinary collaboration.

### Integration with 2D, Topological, and Hybrid Spintronic Systems

Recent advances in spintronics point toward hybrid architectures that combine magnetic oxides with 2D materials (e.g., graphene, MoS_2_), topological insulators (e.g., Bi_2_Se_3_), or organic semiconductors. These systems enable novel phenomena, such as spin momentum locking, gate-tunable magnetism, and room-temperature spin–orbit torque. The low-temperature and solution-processable nature of sol–gel synthesis makes it ideal for forming conformal oxide layers on fragile 2D substrates or flexible organic backbones. For example, ZnO or Fe_3_O_4_ thin films deposited via sol–gel methods can act as spin injectors or filters in MoS_2_-based transistors. Similarly, the integration of sol–gel-derived ferromagnetic oxides with TIs may enable dissipationless edge-state spin transport. In organic–inorganic hybrids, sol–gel processing allows for magnetic nanoparticle embedding into polymer matrices, yielding multifunctional films for wearable or bio-interfaced spintronics. While such integrations remain largely unexplored, they represent a promising frontier for next-generation devices.

## 5. Conclusions

The sol–gel synthesis method has firmly established itself as a versatile and powerful tool in the fabrication of magnetic metal oxide nanostructures for spintronic applications. Its ability to control the composition, dopant dispersion, morphology, and defect states at the molecular level offers unique advantages over traditional solid-state methods. As reviewed in this paper, sol–gel techniques can produce a wide range of nanostructures ranging from nanoparticles and nanorods to thin films and porous monoliths tailored for specific spintronic functionalities, such as spin injection, magnetoresistance, spin filtering, and bio-compatible magnetism. Moreover, the sol–gel method allows for cost-effective, scalable, and environmentally tunable synthesis, enabling researchers to bridge the gap between lab-scale material design and industrial-scale device fabrication. It is especially valuable in developing diluting magnetic semiconductors, ferrites, and perovskites with tunable magnetic properties, making it highly relevant in applications like magnetic tunnel junctions, magnetoresistive sensors, and multifunctional hybrid devices.

While several challenges persist, such as phase separation, inconsistent doping, and defect-induced extrinsic magnetism, ongoing advances in in situ diagnostics, atmosphere-controlled calcination, and machine learning-assisted synthesis optimization are helping overcome these barriers. The method’s adaptability makes it highly suitable for integration with emerging technologies, including 2D materials, flexible substrates, and spin-caloritronic architectures. Looking forward, the sol–gel route is expected to remain at the forefront of magnetic oxide research, driving discoveries in quantum spintronic materials, bio-integrated magnetism, and energy-efficient computing systems. With the incorporation of green chemistry principles and data-driven design, sol–gel synthesis is not only a bridge between chemistry and physics but also a foundational technique for next-generation spintronic innovations. In addition to summarizing the state of sol–gel-based magnetic oxides, this review offers a critical perspective on the field’s most urgent challenges and research opportunities, aiming to inform and inspire future advancements in spintronic material design.

## Figures and Tables

**Table 1:** Advantages and disadvantages of the sol–gel method for synthesizing spintronic oxide nanomaterials [99,100,101].

Aspect	Advantages	Disadvantages	Spintronic Relevance
**Temperature**	Low-temperature processing (<600 °C)	May require post-annealing for full crystallization	Enables compatibility with flexible substrates and CMOS platforms
**Dopant Control**	Atomic-scale mixing ensures uniform dopant distribution	Dopant clustering or segregation may occur	Crucial for tuning magnetic uniformity and room-temperature FM
**Scalability**	Scalable for large-area coatings and batch synthesis	Film uniformity over large substrates is still challenging	Allows wafer-scale or device-scale fabrication of spintronic layers
**Cost**	Uses inexpensive precursors and low-energy processing	Some alkoxides can be air/moisture-sensitive and expensive	Reduces fabrication cost for practical spintronic devices
**Versatility**	Applicable to a wide range of oxides, morphologies, composites	Limited to materials that form stable sol–gel networks	Enables multifunctionality (e.g., magneto-optic, magnetoelectric)
**Defect Engineering**	Facilitates oxygen vacancy control and defect-induced magnetism	Difficult to isolate intrinsic from extrinsic magnetic effects	Key for inducing spin polarization via oxygen vacancies
**Phase Purity**	High compositional homogeneity reduces unwanted phases	Secondary phases (e.g., metallic clusters) may still form	Prevents spurious magnetic signals in spintronic measurements
**Device Compatibility**	Amenable to deposition on silicon, glass, flexible substrates	Drying, shrinkage, and cracking can hinder film quality	Critical for integration into memory and sensor architectures

**Table 2:** Comparative analysis of synthesis methods for spintronic oxide nanomaterials.

Method	Temperature (°C)	Dopant Control	Phase Purity	Scalability	Spintronic Relevance	Cost
**Sol–Gel**	400–600	High (molecular level)	High	High	Good (DMS, ferrites, thin films)	Low
**Hydrothermal**	100–250	Moderate	High	Moderate	Moderate (nanorods, hierarchical)	Moderate
**Solid-State**	>1000	Low	Low	High	Low (poor dopant uniformity)	Low
**CVD**	600–1000	High	High	Low	High (thin films, precise layering)	High

**Table 3:** Comparative summary of sol–gel-synthesized magnetic metal oxides: synthesis approaches, magnetic properties, and spintronic applications.

Oxide System	Dopants	Sol–Gel Variant	Morphology	Magnetic Behavior	Spintronic Relevance
ZnO	Co^2+^	Alkoxide, nitrate-based	Nanoparticles (10–20 nm)	Room-temperature ferromagnetism	DMS, spin filters, photocatalysis
TiO_2_	Mn, Mg	Citrate–nitrate, alkoxide	Thin films, nanocrystals	Defect-mediated FM	Spin filters, magneto-optics
La_1−x_Sr_x_MnO_3_	Dy^3+^	Pechini (polymeric gel)	Porous nanoparticles	Double-exchange FM	Colossal MR, tunnel junctions
NiFe_2_O_4_, CoFe_2_O_4_	Ni^2+^, Co^2+^	Green auto-combustion	Porous spinel powders	Tunable anisotropy, exchange bias	Spin valves, high-frequency devices
CeO_2_	Fe^3+^, Cu^2+^	Citrate-assisted sol–gel	Ultra-fine particles (5–10 nm)	F-center exchange FM	Redox-active spintronic devices

Note: FM = Ferromagnetism; DMS = Dilute Magnetic Semiconductor; and MR = Magnetoresistance.

**Table 4:** Key sol–gel processing parameters and their effects on the magnetic behavior of oxide nanomaterials.

Parameter	Effect on Magnetic Behavior
Calcination temperature	Influences crystallite size, phase formation, and defect structure
Dopant concentration	Controls magnetic ordering, dopant distribution, and potential secondary phases
pH of solution	Affects particle size distribution and gel homogeneity
Atmosphere (air, N_2_, O_2_)	Modulates oxidation state, oxygen vacancies, and defect-induced magnetism

## Data Availability

No new data was created.
