# Correction: Nassar et al. Sol–Gel-Synthesized Metal Oxide Nanostructures: Advancements and Prospects for Spintronic Applications—A Comprehensive Review. *Gels* 2025, *11*, 657

**DOI:** 10.3390/gels12030216

**Published:** 2026-03-06

**Authors:** Kais Iben Nassar, Sílvia Soreto Teixeira, Manuel P. F. Graça

**Affiliations:** I3N-Aveiro, Department of Physics, University of Aveiro, 3810-193 Aveiro, Portugal; silvia.soreto@ua.pt (S.S.T.); mpfg@ua.pt (M.P.F.G.)

In the original publication [1], some in-text citations were found to be insufficiently aligned with the specific statements they were intended to support. These issues were limited exclusively to citation relevance and did not affect the scientific content, data interpretation, results, or conclusions of the review.

In response to the Academic Editor’s recommendations, the authors conducted a comprehensive, fully manual, line-by-line review of the entire manuscript. Some references were removed where they were not technically appropriate (original reference numbers: (8, 11, 16, 17, 25, 28, 33, 35, 39, 41, 48, and 50) [2,3,4,5,6,7,8,9,10,11,12,13], and other citations were replaced with more suitable, authoritative, and field-relevant references. As a result, the reference list was renumbered accordingly.

Following this correction, the references replaced in the published manuscript were (7, 12, 13, 14, 15, 26, 27, 29, 30, 34, 36, 37, 38, 42, 43, 44, 46, 47, 49, 51, 52, 53, 54, 55, 56, 101, 112, and 168) [14,15,16,17,18,19,20,21,22,23,24,25,26,27,28,29,30,31,32,33,34,35,36,37,38,39,40,41] of the original publication, which were replaced by references (7, 10, 11, 12, 13, 21, 22, 23, 24, 27, 28, 29, 30, 32, 33, 34, 36, 37, 38, 39, 40, 41, 42, 43, 44, 89, 100, and 156) [42,43,44,45,46,47,48,49,50,51,52,53,54,55,56,57,58,59,60,61,62,63,64,65,66,67,68,69] in the current version. All these references have been carefully selected to ensure direct relevance to the corresponding statements in the text and have been correctly renumbered throughout the manuscript.

All in-text citations have been manually re-mapped to ensure that each reference now directly and accurately supports the associated statement. All remaining references were thoroughly verified and confirmed to be relevant and technically justified.

No changes were made to the manuscript text, figures, tables, scientific interpretations, results, or conclusions. The scope, structure, and scientific content of the review remain identical to the originally published version. All corrections are strictly limited to citation relevance, accuracy, and traceability.

This correction was approved by the Academic Editor. The original publication has been updated accordingly.
